# Effectiveness of Protein-enriched oral nutritional supplements on muscle function in middle-aged and elderly women: A randomized controlled trial

**DOI:** 10.1016/j.jnha.2025.100508

**Published:** 2025-02-13

**Authors:** Minji Kang, Hyunkyung Rho, Minhui Kim, Miji Lee, Yunsook Lim, Jinmann Chon, Hyunjung Lim

**Affiliations:** a Department of Medical Nutrition, Graduate School of East-West Medical Science, Kyung Hee University, Giheong-gu, Yongin 17104, Republic of Korea; b Research Institute of Medical Nutrition, Kyung Hee University, Dongdaemoon-gu, Seoul 02447, Republic of Korea; c Department of Food and Nutrition, Kyung Hee University, Dongdaemoon-gu, Seoul 02447, Republic of Korea; d Department of Rehabilitation Medicine, Kyung Hee University Medical Center, Dongdaemun-gu, Seoul 02447, Republic of Korea

**Keywords:** Oral nutritional supplements, Protein-enriched ONS, Muscle function, Lean body mass, Branched chain amino acid, Sarcopenia

## Abstract

**Objectives:**

This study evaluated the effects of protein-enriched oral nutritional supplementation (ONS) consumption for 12 weeks on muscle mass, muscular strength, and function in middle-aged and elderly women.

**Design:**

A single-center, double-blind, randomized controlled trial

**Participants:**

This study was conducted with 70 healthy female participants aged 50–80.

**Intervention and measurements:**

Participants were instructed to incorporate two daily packs of either the test ONS (Nucare Active, Daesang Wellife Corp., Seoul, Republic of Korea; 200 kcal/pack, 23 g carbohydrate, 6 g fat, and 15 g protein including branched chain amino acids or placebo ONS (200 kcal/pack, 33 g carbohydrate, 8 g fat, and 1 g protein) into their routine for 12 weeks while maintaining their regular lifestyle. The primary outcome was lean body mass (LBM), while secondary outcomes included muscular strength, physical performance ability, inflammatory markers, and body fat mass (FM).

**Results:**

Sixty-four participants (33 in the test group, 31 in the placebo group; mean ± SD age, [test] 63.06 ± 5.51 years, [placebo] 63.29 ± 3.28 years, *p* = 0.839) completed the 12-week protocol. The test group exhibited a higher percentage change in LBM than the placebo group (0.26 % [95%CI: −0.27, 0.78] vs. −0.47 % [95%CI: −0.81, −0.13]; *p* = 0.020). The placebo group experienced a significant increase in FM (38.15 % [95%CI: 36.62, 39.69] to 38.67 % [95%CI: 37.14, 40.21]; *p* < 0.01). The difference in the changes in LBM/BMI and FM/BMI between the two groups was also visually distinct. There were no significant differences between the two groups in terms of muscular strength, physical performance ability, or inflammatory markers.

**Conclusions:**

Protein-enriched ONS helped maintain LBM and prevent FM gain in middle-aged and elderly females. This suggests its potential role in preventing frailty and musculoskeletal disorders associated with female aging.

## 1 Introduction

Sarcopenia is the loss of muscle mass and strength that occurs with aging [1]. The European Working Group on Sarcopenia in Older People 2 (EWGSOP2) defined severe sarcopenia as detecting all low muscle strength, low muscle quantity/quality, and low physical performance [2]. Histologically, loss of skeletal muscle mass and strength with aging is characterized by a selectively reduced size and greater atrophy of type 2 fibers that support high intensity exercise [3]. Furthermore, high fatness predicts loss of muscle mass and is associated with muscle quality [4]. Sarcopenia leads to reduced mobility, resulting in a decline in quality of life and increasing the risk of fall-related injuries [5]. This can lead to prolonged hospitalization and rehabilitation, imposing significant healthcare burdens [5]. The loss of skeletal muscle mass progresses gradually at a rate of 0.5%–1% annually, accelerating beyond the age of 60, underscoring the need for preventive management from middle age onwards [6]. Nevertheless, while the emphasis on the need for management from middle age is underscored, research on muscle loss and sarcopenia mostly focuses on individuals aged 65 and older [7].

Women in middle age and beyond are known to experience significantly greater declines in muscle mass and force-generating capacity of muscle compared to men of the same age group [8]. Additionally, women tend to experience an increase in visceral fat and a decrease in muscle mass and strength as they age [9]. This can be explained by the influence of estrogen on protein synthesis/degradation signaling pathways, apoptotic signaling pathways, and contractile protein modifications [8]. Therefore, preventive management of muscle mass decline after middle age is emphasized more in women compared to men.

The cause of sarcopenia can be a decrease in exercise and changes in sex hormone, growth hormone, and inflammatory cytokines [[10], [11], [12], [13]]. Inadequate nutrition and malabsorption among the mechanisms that can contribute to the onset and progression of sarcopenia [14]. Interventions targeting modifiable lifestyle factors such as dietary modifications are a promising approach for preventing sarcopenia and preserving muscle mass [15]. Studies have suggested that supplementation of nutrients such as protein and vitamins may be helpful in preventing sarcopenia and improving muscle mass and strength [[16], [17], [18], [19], [20]]. However, despite observational findings suggesting that protein or other nutrient supplementation may support improvements in muscle mass and function, the overall level of evidence remains limited. The effects of additional protein or nutrient intake alone have shown inconsistent results, except in individuals with low habitual intake or malnutrition. Therefore, further research is needed to determine whether such supplementation can be beneficial even in healthy individuals without clinical malnutrition. Additionally, most of these studies have focused on individual nutrients, whereas the combined effects of multiple nutrients may be more effective. Since individuals consume various nutrients together rather than in isolation, investigating the impact of multi-nutrient supplementation, such as oral nutritional supplements (ONS), may provide more meaningful insights.

ONS are beverages designed to provide nutrients conveniently, particularly for individuals who may not consume adequate nutrition through regular meals. They come in various types tailored to specific patient needs or nutritional goals, offering enhanced levels of particular nutrients or addressing specific dietary requirements. If it is difficult to meet their nutritional requirements through meals, ONS, which is designed to be supplied through oral or tube feeding, can be used. This study aimed to evaluate the effects of protein-enriched ONS on muscle mass, muscular strength, and function in healthy females aged 50–80 years.

## 2 Methods

### 2.1 Study design

This prospective, single-center, double-blind, randomized controlled trial was conducted from February 19, 2020, to September 23, 2021, in Kyung Hee University Medical Center (Seoul, Republic of Korea). This study was approved by the Institutional Review Board (IRB) of Kyung Hee University Medical Center (IRB No. KHUH 2019-12-041) and registered at Clinical Research Information Service (CRIS; https://cris.nih.go.kr/cris/index/index.do) (CRIS No. KCT0007708). This study follows the Consolidated Standards of Reporting Trials (CONSORT) reporting guideline.

**Fig. 1:**
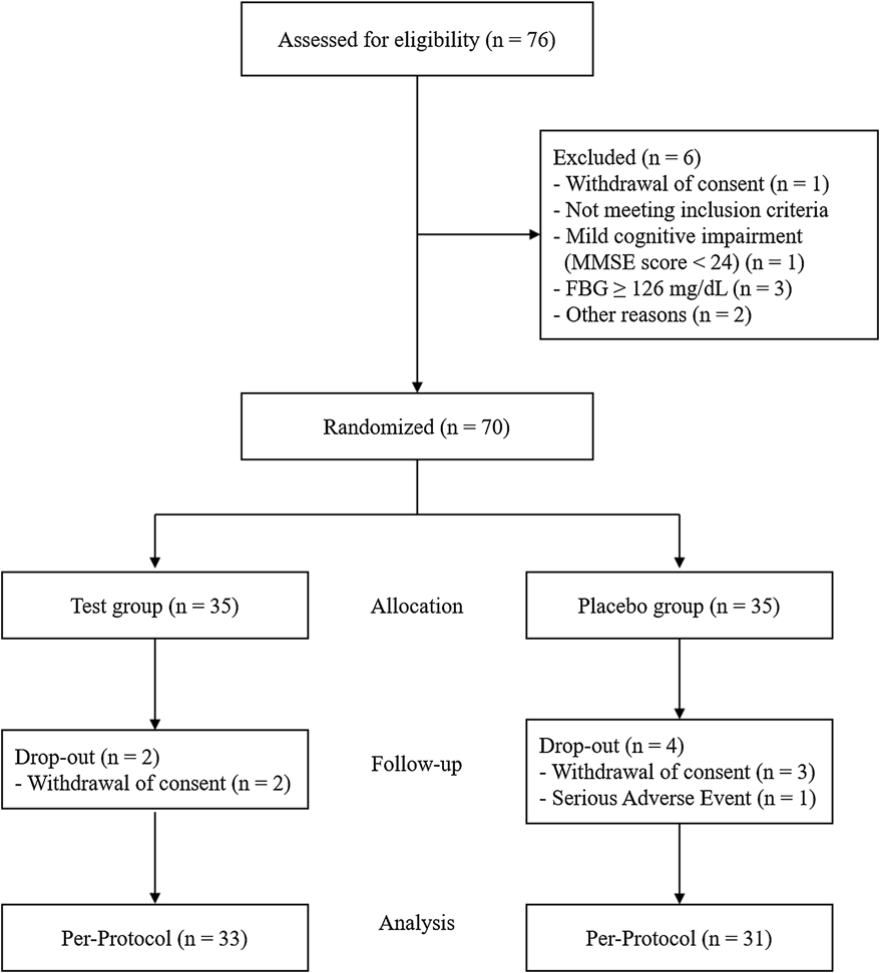
Flow diagram of study participants.

All participants provided written consent prior to the start of the study. Participants were randomly assigned 1:1 to the test and placebo groups using a probability-based randomization method (www.randomization.com) (Fig. 1). Participants were assigned based on lower numbers following the randomized table in the order of their visit date. Each participant received a test product or placebo product corresponding to their assigned number. Blinding was maintained for all participants, researchers, and outcome assessors until the end of the trial, after which the blinding was lifted.

Participants were instructed to consume an additional two packs of ONS (200 ml, 200 kcal per pack) daily for 12 weeks. The two packs of ONS were intended to be consumed in the evening. Throughout the study period, they were directed to maintain their regular daily routines and dietary intake. Participants completed a 3-day food record for dietary assessment, and physical activity was evaluated using the Korean version of the international physical activity questionnaire (IPAQ) [21]. Efficacy indicators were evaluated at baseline and at week 12. Adverse events were assessed at weeks 6 and 12.

### 2.2 Participants

The eligibility criteria for the study population included adult women aged 50–80 years. The exclusion criteria were as follows: ‘body mass index (BMI) ≥ 30 kg/m^2^’, ‘a diabetes mellitus or hypertension patient who cannot be controlled by taking medication’, ‘renal or liver dysfunction’, ‘cognitive disorder or mental illness’, ‘serious musculoskeletal problems’, ‘unable to walk’, ‘diagnosed with acute or serious disease (liver disease, kidney disease, heart disease, thyroid disease or cancer) or taking medication’, ‘continue to perform aerobic or resistance exercise within 6 months’, ‘continuously taking hormones, muscle strengthening agents or functional food related to protein supplementation, muscle function improvement, and exercise performance within 3 months’, ‘history of surgery within 6 months’, ‘drinking more than 140 g of alcohol per week’, ‘history of drug addiction or alcoholism’, ‘allergy or hypersensitive against ingredients of the test product’, ‘participating in another clinical trial within a month’, ‘unable to consume the investigational product (ONS) during the study period’, ‘illiterate’, ‘pregnancy or breastfeeding’, and for other reasons the researcher determine to be unsuitable for participation in this study.

### 2.3 Investigational products

Two investigational products, each containing 200 kcal per pack, were compared. The test ONS (Nucare Active, Daesang Wellife Corp., Seoul, Republic of Korea), developed to prevent and improve sarcopenia, contains protein (15 g per pack) and includes branched-chain amino acids (BCAAs; 2000 mg per pack) as the main components. Additionally, it is nutritionally balanced with a macronutrient ratio of 43:30:27 (carbohydrates 23 g, protein 15 g, fat 6 g). On the other hand, the placebo ONS contains less protein compared to the test ONS and does not include BCAAs. Each pack of the placebo ONS contains 33 g of carbohydrates (64% of energy), 1 g of protein (1% of energy), and 8 g of fat (35% of energy).

### 2.4 Primary outcome

The primary outcome was muscle mass measured by dual-energy X-ray absorptiometry (DXA; Lunar iDXA, GE Healthcare, Chicago, IL, USA). DXA was conducted at week 0 and endpoint (week 12) after at least 8 h of fasting. The measured variable was total lean body mass (kg or %).

### 2.5 Secondary outcome

The secondary outcomes were muscular strength, physical performance ability, inflammatory markers, and body fat mass. Muscular strength was evaluated by handgrip strength (HGS), knee extension strength (KES), and knee flexor strength (KFS). Physical performance ability was evaluated using usual gait speed (UGS), short physical performance battery (SPPB), and timed up and go test (TUG). Inflammatory markers included interleukin-6 (IL-6), tumor necrosis factor-α (TNF-α), and high-sensitivity C-reactive protein (hs-CRP). Body fat mass (kg or %) was measured using DXA.

#### 2.5.1 Handgrip strength (HGS)

The subjects sat in a chair, bent the elbow at 90 °, aligned the handle of the dynamometer (Jamar Plus Digital hand dynamometer, Paterson Medical, Green Bay, WI, USA) with the second knuckle of the finger, and then grasped the handle as tightly as possible [22]. HGS was measured alternately with the left and right hands 3 times each, and the maximum value was recorded at up to 0.1 kg.

#### 2.5.2 Knee extensor strength (KES) and knee flexor strength (KFS)

Leg muscle strength (KES and KFS) tests were conducted using the leg muscle dynamometer (IB-LS; InBody Co. Ltd., Seoul, Republic of Korea). The subjects sat on the leg muscle dynamometer with their hips reaching the backrest and holding the handle. Then, the legs were fixed with the fixing bar of the device, and the leg muscle strength of both legs was measured twice, and the maximum value was recorded at up to 0.1 kg. The absolute KES was the value that calculated the sum of the maximum left and right KES. The absolute KFS was the value that calculated the sum of the maximum left and right KFS.

#### 2.5.3 Usual gait speed (UGS)

The subjects wore comfortable clothes and shoes and walked a distance of 7 meters, including 1.5 meters in each acceleration and deceleration section at a usual speed. The subjects were asked to walk 7 meters from the starting point and then stop. At the end of the 1.5 meters acceleration section, the trained researcher started measurement by pressing the stopwatch when the toe of the subject's forefoot passed and pressed the stopwatch again at the end of 4 meters section to measure the time. The measured time was recorded in two decimal places, and the test was repeated twice, and the average value was used [23]. The UGS was the average value (seconds) divided by 4 meters.

#### 2.5.4 Short physical performance battery (SPPB)

SPPB test included walking speed, standing balance, and ability to rise from a chair. The SPPB was scored on a scale of 0–12, with 0 being the lowest and 12 being the highest [24].

#### 2.5.5 Timed up and go test (TUG)

The TUG test is mainly performed as a screening test for gait abnormalities. The subject sat comfortably in a chair, stood up, moved forward 3 m, and then came back to sit down. The time from the moment the subject got up from the chair to the moment they completed the procedure and sat down was measured. If it took more than 13.5 seconds, the possibility of a fall was evaluated [25].

#### 2.5.6 Inflammatory markers

Blood samples were collected from participants who had fasted for at least 8 h and were centrifuged at 3,000 rpm for 10 min in a serum separation tube and an ethylenediaminetetraacetic acid tube. IL-6 and TNF-α were analyzed by enzyme-linked immunosorbent assay kits. The hs-CRP level was analyzed using a turbidimetric immunoassay kit. All procedures were performed according to the manufacturer's instructions.

### 2.6 Compliance

The compliance evaluation was conducted twice in total (week 6 and week 12). ONS was delivered to participants twice in total, at baseline and at week 6, via courier. To evaluate participants' compliance with ONS intake, participants were instructed to return any unused ONS at the expected consumption completion points (i.e., week 6 and week 12). During each visit (week 6 and week 12), the researchers counted the number of remaining ONS products. The subjects with less than 80% compliance on two consecutive occasions were excluded from the analysis. Compliance was calculated as follows: (number of the ONS actually consumed/number of the ONS to be consumed) × 100.

### 2.7 Adverse events

Adverse events refer to undesirable and unintended signs, symptoms, or diseases that occur after the consumption of the investigational product (ONS). However, they do not necessarily imply a causal relationship with the product. If an event considered as a potential adverse reaction is observed during the study, all relevant details, including symptoms, onset date, and duration, are thoroughly documented. The study investigator assesses the severity of the adverse event, and if necessary, the association with the investigational product is further evaluated with the opinion of the responsible medical staff.

### 2.8 Statistical methods

Sample size calculation was conducted in a study that investigated the effect of exercise and supplementation of amino acid supplements on physical activity ability in women with sarcopenia [26]. A paired t-test at a significance level of 5% and a power of 0.85 based on a 95% confidence interval was used. The total number of subjects was 58, including 29 per group, and the final number of subjects was set at 70 based on a 17% dropout rate.

The per-protocol set was analyzed for subjects who had acclimatized to a certain level or more and completed the test. Continuous variables were expressed as mean ± standard deviation (SD) or mean (95% confidence interval [CI]) [% coefficient of variation (CV)]. Categorical variables were expressed as number (%). The χ^2^ test and student's t-test (or Mann-Whitney test) were performed to compare the test group and the placebo group at week 0, at week 12, and the amount of change. A paired t-test or Wilcoxon signed-rank test was performed to compare week 0 and week 12 values within the group. ANCOVA was performed with initial factors that showed differences between groups as covariates. The statistical analysis was conducted using the SAS version 9.4 (SAS Institute, Cary, NC, USA). The significance level was set at a *p* < 0.05.

## 3 Results

Out of 70 participants, 64 (31 placebo group and 33 test group) completed the protocol over the 12-week period and were included in the analysis (Fig. 1). There were no significant differences in compliance between the two groups (placebo, 97.20 ± 5.48 %; test, 98.30 ± 2.84 %; *p* = 0.297). The adverse events that occurred during the study were 3 cases in the placebo group (1 case each of lung cancer, wrist sprain, and hyperlipidemia) and 1 case in the test group (Helicobacter pylori infection), totaling 4 cases. These events were confirmed to have no clear association with the study. In both groups, no participant exhibited compliance below 80% for two consecutive assessments.

The comparison of demographic and health-related characteristics between the two groups is presented in Table 1. Except for baseline physical activity levels, there were no significant differences between the groups. At baseline, there was a significant difference in physical activity levels between the test and placebo groups due to individual variations (placebo, 1894.82 ± 1307.15 MET-min/week; test, 2989.38 ± 1904.20 MET-min/week; *p* = 0.009), but after 12 weeks, there were no significant changes in physical activity within each group (placebo, Δ 119.89 ± 1136.29, *p* = 0.561; test, Δ −418.55 ± 1622.78, *p* = 0.148). This indicates that habitual lifestyle was maintained without changes in physical activity levels over the course of 12 weeks. Furthermore, there was no significant difference between the two groups after 12 weeks (placebo, 2014.71 ± 1383.42 MET-min/week; test, 2570.83 ± 1569.94 MET-min/week; *p* = 0.139). Despite the additional supplementation of ONS, there was no change in total energy intake for both groups before and after 12 weeks (Table 2). There were no significant differences in nutrient intake between the two groups at baseline. The differences in the nutrient composition of the two ONS resulted in an increase in protein, BCAAs, calcium, phosphorus, vitamin D, E, and C intake in the test group after 12 weeks.

**Table 1:** Demographic and health-related characteristics of middle-aged and elderly women at baseline.

Variables	Placebo (n = 31)	Test (n = 33)	*p*^2^
Age (years)	63.29 ± 3.28^1^	63.06 ± 5.51	0.839
Height (cm)	154.39 ± 4.03	155.02 ± 5.43	0.603
Weight (kg)	58.93 ± 6.63	56.75 ± 7.01	0.207
Body mass index (kg/m^2^)	24.76 ± 2.89	23.58 ± 2.39	0.077
Systolic blood pressure (mmHg)	124.90 ± 12.90	122.76 ± 12.27	0.498
Diastolic blood pressure (mmHg)	78.71 ± 8.77	76.70 ± 6.78	0.307
Marriage status (n [%])			
Yes	2 (6.45)	1 (3.03)	0.607
No	29 (93.55)	32 (96.97)	
Disease history (n [%])			
Yes	20 (64.52)	19 (57.58)	0.616
No	11 (35.48)	14 (42.42)	
Alcohol drinking (n [%])			
Yes	7 (22.58)	11 (33.33)	0.410
No	24 (77.42)	22 (66.67)	
Smoking (n [%])			
Yes	0 (0.00)	0 (0.00)	1.000
No	31 (100.00)	32 (96.97)	
Ex-smoker	0 (0.00)	1 (3.03)	
Physical activity (MET-min/week)^3^			
Baseline	1894.82 ± 1307.15	2989.38 ± 1904.20	**0.009**
After 12 weeks^4^	2014.71 ± 1383.42	2570.83 ± 1569.94	0.139

1 Values are expressed as means ± standard deviation.

2 P values were obtained from Fisher's extract for categorical variables and student's t-test for continuous variables.

3 Levels of physical activity were measured with the Korean version of the international physical activity questionnaire, and calculated as the metabolic equivalent of task (MET)-minute/week.

4 Significant difference between baseline and 12 weeks data by paired t test at * *p* < 0.05, ** *p* < 0.01, *** *p* < 0.001.

**Table 2:** Dietary intake of middle-aged and elderly women at baseline and after 12 weeks.^1^

	Placebo (n = 31)		Test (n = 33)			
Variables	Baseline	12 weeks^2^^,^^3^	Baseline	12 weeks^2^^,^^3^	*p*^4^	*p*^5^
Energy (kcal)	1736.40 ± 348.25^6^	1774.16 ± 316.84	1762.04 ± 356.48	1761.82 ± 378.01	0.772	0.888
C:P:F ratio (%)	59:16:25	60:13:27	59:16:25	56:19:25		
Proteins (g)	68.68 ± 18.31	57.35 ± 16.11**	69.48 ± 17.36	85.76 ± 18.63***	0.857	**<.0001**
Protein intake per body weight (g/kg)	1.17 ± 0.29	0.98 ± 0.29**	1.23 ± 0.29	1.54 ± 0.38***	0.391	**<.0001**
BCAAs (mg)						
Isoleucine (mg)	2133.32 ± 1405.19	1346.16 ± 515.88**	1730.98 ± 701.53	2287.67 ± 692.31***	0.158	**<.0001**
Leucine (mg)	3298.68 ± 1318.08	2439.52 ± 969.47**	3105.08 ± 1211.33	4350.24 ± 1231.68***	0.543	**<.0001**
Valine (mg)	2127.06 ± 807.75	1548.42 ± 544.61**	2049.45 ± 767.15	2488.29 ± 727.49**	0.695	**<.0001**
Carbohydrates (g)	253.03 ± 44.21	266.39 ± 43.61	258.97 ± 56.14	245.22 ± 60.23	0.641	0.114
Fiber (g)	25.33 ± 6.91	26.90 ± 6.59	26.09 ± 8.09	27.43 ± 6.99	0.690	0.759
Fats (g)	47.73 ± 19.68	53.70 ± 13.98	48.13 ± 18.64	49.54 ± 15.05	0.934	0.256
Cholesterol (mg)	314.21 ± 144.20	317.33 ± 214.99	327.96 ± 181.89	292.64 ± 130.55	0.740	0.584
Saturated fatty acid (g)	10.64 ± 6.13	12.70 ± 5.54*	8.98 ± 4.07	11.55 ± 5.02**	0.213	0.386
Calcium (mg)	568.14 ± 176.92	738.92 ± 197.03***	628.66 ± 243.00	1149.33 ± 196.45***	0.262	**<.0001**
Phosphorus (mg)	1053.97 ± 246.29	1156.09 ± 257.22	1165.23 ± 314.58	1497.63 ± 291.28***	0.122	**<.0001**
Sodium (mg)	3395.64 ± 1038.20	3228.78 ± 1046.81	3461.86 ± 1149.97	3225.29 ± 1078.44	0.810	0.990
Potassium (mg)	2675.10 ± 561.26	2958.69 ± 650.16*	3271.48 ± 1149.10	3280.95 ± 938.04	0.011	0.114
Magnesium (mg)	116.76 ± 37.37	243.50 ± 36.49***	113.62 ± 48.57	242.70 ± 39.33***	0.774	0.933
Iron (mg)	16.80 ± 5.86	18.69 ± 4.34	17.25 ± 5.73	18.97 ± 7.65	0.757	0.854
Zinc (mg)	9.90 ± 2.83	12.66 ± 2.77***	9.68 ± 2.69	24.61 ± 2.70***	0.751	**<.0001**
Vitamin A (μg RAE)	461.03 ± 169.97	755.07 ± 244.58***	552.41 ± 415.33	1051.42 ± 184.12***	0.251	**<.0001**
Vitamin D (μg)	4.81 ± 6.77	5.57 ± 3.85	4.59 ± 5.31	29.37 ± 3.39***	0.885	**<.0001**
Vitamin E (mg α-TE)	15.70 ± 5.77	18.15 ± 5.87	16.49 ± 7.74	28.17 ± 5.59***	0.650	**<.0001**
Vitamin C (mg)	145.21 ± 68.73	171.57 ± 92.94	125.57 ± 71.21	182.55 ± 36.14***	0.266	0.542
Thiamine (mg)	1.67 ± 0.43	1.83 ± 0.43*	1.74 ± 0.51	1.83 ± 0.51	0.535	0.948
Riboflavin (mg)	1.48 ± 0.35	1.70 ± 0.45*	1.42 ± 0.39	1.74 ± 0.35***	0.459	0.727
Niacin (mg NE)	13.50 ± 3.89	16.05 ± 3.31**	13.36 ± 4.56	16.58 ± 3.92***	0.898	0.562
Vitamin B_6_ (mg)	1.63 ± 0.39	2.34 ± 1.75*	2.20 ± 1.44	2.06 ± 0.87	0.037	0.414
Folate (μg)	501.87 ± 126.48	644.51 ± 200.61***	560.28 ± 213.30	552.97 ± 129.39	0.185	0.036
Vitamin B_12_ (μg)	7.00 ± 4.38	7.30 ± 3.48	8.06 ± 5.07	7.08 ± 3.18	0.376	0.791
Pantothenic acid (mg)	4.19 ± 1.19	5.32 ± 1.17***	4.27 ± 1.26	5.39 ± 1.05***	0.812	0.798
Biotin (μg)	2.71 ± 1.83	14.89 ± 2.75***	3.55 ± 3.22	15.95 ± 4.27***	0.206	0.237

C, carbohydrates; P, protein; F, fat; RAE, retinol activity equivalent; TE, tocopherol equivalent; NE, nicotinic acid equivalent; BCAAs, branched chain amino acids.

1 Dietary intake was assessed through 3-day food records. The 3-day food records include 2 weekdays and 1 weekend. The intake of provided oral nutritional supplementation was also written in the 3-day food record at week 12.

2 The dietary intake including oral nutritional supplementation.

3 Significant difference between baseline and 12 weeks data by paired t test at * *p* < 0.05, ** *p* < 0.01, *** *p* < 0.001.

4 Significant difference at baseline between the groups by student t-test.

5 Significant difference at 12 weeks between the groups by student t-test.

6 Values are expressed as means ± standard deviation.

The changes in muscle mass and body fat mass of the participants after 12 weeks are presented in Table 3. The change in lean body mass after 12 weeks was higher in the test group compared to the placebo group (test, Δ 0.26 % [95%CI: −0.27, 0.78; %CV: 578.5]; placebo, Δ −0.47 [95%CI: −0.81, −0.13; %CV: −196.3]; *p* = 0.020). The ASM adjusted for AFM exhibited a significant increase after 12 weeks in the test group (1.74 kg/kg [95%CI: 1.65, 1.83; %CV: 14.1] to 1.80 kg/kg [95%CI: 1.70, 1.91; %CV: 16.4]; *p* < 0.05), while the placebo group showed a decrease (placebo, Δ −0.02 kg/kg [95%CI: −0.05, 0.01; %CV: −364.8]; test, Δ 0.06 kg/kg [95%CI: 0.01, 0.10; %CV: 215.9]; *p* = 0.003). There was no change in body weight and body fat mass in the test group, whereas an increase was observed in the placebo group (weight, 58.93 kg [95%CI: 56.50, 61.36; %CV: 11.3] to 59.41 kg [95%CI: 56.88, 61.94; %CV: 11.6], *p* < 0.05; body fat mass, 21.58 kg [95%CI: 19.97, 23.19; %CV: 20.3] to 22.19 kg [95%CI: 20.52, 23.86; %CV: 20.5], *p* < 0.01).

**Table 3:** Changes in muscle mass and body fat mass of middle-aged and elderly women after 12 weeks.

Variables	Placebo (n = 31)			Test (n = 33)			*p*^2^^,^^3^
	Baseline Mean (95%CI) [%CV]	12 weeks^1^ Mean (95%CI) [%CV]	Δ 12 wk-baseline Mean (95%CI) [%CV]	Baseline Mean (95%CI) [%CV]	12 weeks^1^ Mean (95%CI) [%CV]	Δ 12 wk-Baseline Mean (95%CI) [%CV]	
Weight (kg)	58.93 (56.50, 61.36) [11.3]	59.41* (56.88, 61.94) [11.6]	0.48 (0.01, 0.92) [249.9]	56.75 (54.27, 59.24) [12.4]	56.65 (56.65, 54.16) [12.4]	−0.11 (−0.43, 0.22) [−870.2]	**0.032**
Fat mass							
Body fat (kg)	21.58 (19.97, 23.19) [20.3]	22.19** (20.52, 23.86) [20.5]	0.61 (0.29, 0.94) [142.8]	20.03 (18.50, 21.56) [21.6]	19.93 (18.34, 21.58) [22.9]	−0.07 (−0.40, 0.27) [−1403.7]	0.003*
Body fat (%)^4^	38.15 (36.62, 39.69) [10.9]	38.67** (37.14, 40.21) [10.8]	0.52 (0.16, 0.88) [187.8]	36.68 (35.17, 38.19) [11.6]	36.43 (34.72, 38.14) [13.2]	−0.25 (−0.79, 0.29) [−603.9]	**0.018**
AFM (kg)	8.89 (8.25, 9.53) [19.7]	9.12** (8.45, 9.78) [19.8]	0.23 (0.07, 0.38) [185.6]	8.42 (7.88, 8.95) [18.0]	8.23** (7.71, 8.76) [18.1]	−0.18 (−0.31, −0.06) [−193.3]	<.0001***
Muscle mass							
Lean body mass (kg)	34.50 (33.51, 35.49) [7.8]	34.73 (33.61, 35.84) [8.8]	0.23 (−0.04, 0.50) [324.5]	34.12 (32.90, 35.34) [10.1]	34.31 (33.13, 35.49) [9.7]	0.19 (−0.10, 0.48) [430.1]	0.847
Lean body mass (%)^5^	59.84 (58.39, 61.29) [6.6]	59.37 (57.93, 60.82) [6.6]	−0.47 (−0.81, −0.13) [−196.3]	61.19 (59.77, 62.61) [6.5]	61.44 (59.84, 63.05) [7.4]	0.26 (−0.27, 0.78) [578.5]	**0.020**
ASM (kg)	14.59 (14.12, 15.06) [8.8]	14.78* (14.25, 15.31) [9.8]	0.19 (0.04, 0.34) [212.8]	14.40 (13.73, 15.07) [13.1]	14.52 (13.86, 15.19) [12.9]	0.12 (−0.02, 0.26) [322.8]	0.504
ASM/BMI (kg/kg/m^2^)	0.59 (0.57, 0.61) [8.5]	0.60 (0.58, 0.62) [8.9]	0.00 (0.00, 0.01) [434.9]	0.61 (0.59, 0.63) [10.6]	0.62* (5.39, 5.61) [5.8]	0.01 (0.00, 0.01) [262.7]	0.338
ASM/AFM (kg/kg)	1.69 (1.58, 1.80) [17.5]	1.67 (1.56, 1.78) [17.7]	−0.02 (−0.05, 0.01) [−364.8]	1.74 (1.65, 1.83) [14.1]	1.80* (1.70, 1.91) [16.4]	0.06 (0.01, 0.10) [215.9]	0.003***

AFM, appendicular fat mass; ASM, appendicular skeletal muscle mass; BMI, body mass index; CI, confidence interval; CV, coefficient of variation.

1 Significant difference between baseline and 12 weeks data by paired t test at * *p* < 0.05, ** *p* < 0.01, *** *p* < 0.001.

2 Significant difference in changes between baseline and 12 weeks by student t-test.

3 Significant difference in changes between baseline and 12 weeks by ANCOVA adjusted for physical activity level at * *p* < 0.05, ** *p* < 0.01, *** *p* < 0.001.

4 Body fat (%) was calculated as body fat mass (kg) / (fat mass (kg) + lean body mass (kg) + bone mineral content (kg)) * 100.

5 Lean body mass (%) was calculated as lean body mass (kg)/(fat mass (kg) + lean body mass (kg) + bone mineral content (kg)) * 100.

**Fig. 2:**
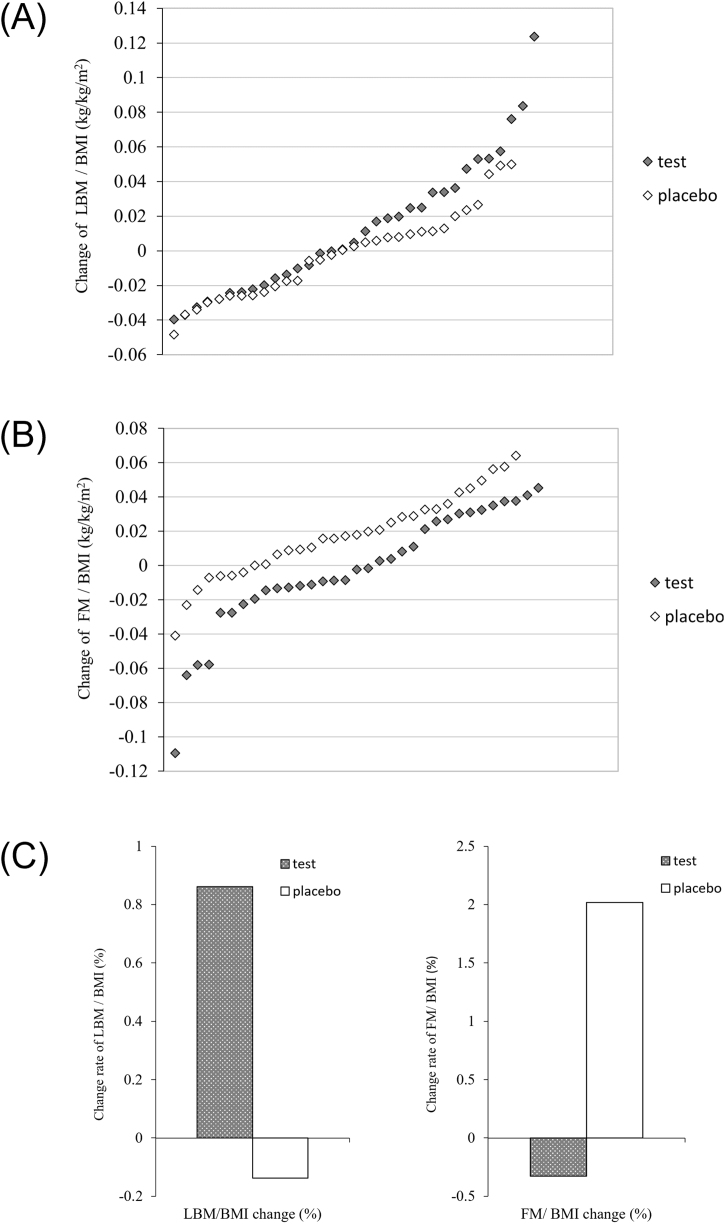
Change of BMI-adjusted lean body mass and BMI-adjusted body fat by group. (A) The change in LBM adjusted for individual BMI within each of the two groups. (B) The change in FM adjusted for individual BMI within each of the two groups. (C) Comparison of the mean change rate in BMI-adjusted LBM and BMI-adjusted FM between the groups. (A) and (B) represent the individual participants' change values arranged in ascending order based on the magnitude of change. Each diamond marker represents the change value of an individual participant. LBM, lean body mass; BMI, body mass index; FM, fat mass.

Fig. 2 lists the changes in lean body mass and fat mass values before and after the trial, adjusting for individual BMI (divided by BMI) to exclude bias caused by BMI [27,28]. The difference in mean changes between the two groups is also indicated. Upon arranging the changes in lean body mass and fat mass for each participant in order of magnitude, it was observed that the test group tended to exhibit greater individual changes in lean body mass lean body mass (adjusted for BMI) compared to the placebo group (Fig. 2A), while changes in fat mass (adjusted for BMI) tended to be smaller in the test group than in the placebo group (Fig. 2B). The average changes for each group showed that the test group exhibited an increase in lean body mass and a decrease in fat mass compared to the placebo group, which showed a decrease in lean body mass and an increase in fat mass (Fig. 2C).

The changes in muscular strength, physical performance ability, and inflammatory markers after 12 weeks for the participants are presented in Table 4. While there was no significant difference in HGS after 12 weeks in both groups, both groups showed a significant increase in KES and KFS. In terms of physical performance ability, both groups showed an increase in SPPB scores, but there was no significant difference in the change between the two groups. The inflammatory markers showed no significant difference between the two groups.

**Table 4:** Changes in muscular strength, physical performance ability, and inflammatory markers of middle-aged and elderly women after 12 weeks.

Variables	Placebo (n = 31)			Test (n = 33)			*p*^2^^,^^3^
	Baseline Mean (95%CI) [%CV]	12 weeks^1^ Mean (95%CI) [%CV]	Δ 12wk-baseline Mean (95%CI) [%CV]	Baseline Mean (95%CI) [%CV]	12 weeks^1^ Mean (95%CI) [%CV]	Δ 12wk-baseline Mean (95%CI) [%CV]	
Muscular strength							
Hand grip strength (kg)	25.15 (23.66, 26.63) [16.1]	24.78 (23.50, 26.05) [14.0]	−0.37 (−1.04, 0.30) [−496.0]	25.45 (24.13, 26.78) [14.7]	25.72 (24.42, 27.02) [14.2]	0.27 (−0.33, 0.86) [631.5]	0.153
Absolute knee extensor strength (kg)	52.60 (48.29, 56.90) [22.3]	57.25** (52.60, 61.89) [22.1]	4.65 (1.48, 7.81) [185.6]	57.04 (51.39, 62.69) [27.9]	61.38** (55.65, 66.11) [21.8]	4.34 (1.20, 7.47) [203.8]	0.887
Absolute knee flexor strength (kg)	33.37 (30.40, 36.34) [24.3]	37.29* (34.70, 39.87) [18.9]	3.92 (1.00, 6.84) [203.2]	38.22 (34.13, 42.32) [30.2]	42.85** (39.49, 46.22) [22.1]	4.63 (1.85, 7.41) [169.1]	0.719
Physical performance ability							
Usual gait speed (sec)	2.80 (2.71, 2.90) [9.07]	2.81 (2.70, 2.93) [11.2]	0.01 (−0.09, 0.11) [2540.5]	2.72 (2.62, 2.83) [10.9]	2.65 (2.55, 2.75) [10.5]	−0.07 (−0.16, 0.01) [−327.0]	0.190
Timed up and go test (sec)	7.79 (7.43, 8.14) [12.5]	7.92 (7.65, 8.20) [9.5]	0.14 (−0.17, 0.44) [611.6]	7.99 (7.68, 8.30) [11.0]	7.94 (7.62, 8.25) [11.2]	−0.05 (−0.33, 0.24) [−1628.6]	0.368
Short physical performance battery (score)	11.48 (11.22, 11.75) [6.3]	11.87** (11.75, 12.00) [2.9]	0.39 (0.16, 0.61) [158.9]	11.76 (11.58, 11.94) [4.3]	11.97* (11.91, 12.03) [1.5]	0.21 (0.04, 0.38) [228.5]	0.210
Inflammatory markers							
IL-6 (pg/mL)	1.78 (0.95, 2.61) [126.8]	1.68 (0.90, 2.46) [126.9]	−0.10 (−0.32, 0.11) [−562.9]	2.52 (1.23, 3.81) [144.2]	2.58 (0.63, 4.54) [213.5]	0.06 (−0.88, 1.00) [4165.9]	0.726
TNF-α (pg/mL)	4.89 (3.88, 5.90) [56.4]	4.13 (3.52, 4.73) [40.0]	−0.77 (−1.90, 0.37) [−403.2]	4.28 (3.52, 5.03) [49.8]	5.45 (3.30, 7.59) [111.1]	1.17 (−1.05, 3.40) [535.2]	0.120
hs-CRP (mg/L)	1.18 (−0.12, 2.48) [300.5]	0.62 (0.40, 0.85) [98.3]	−0.56 (−1.86, 0.75) [−639.9]	0.72 (0.34, 1.10) [150.0]	0.69 (0.40, 0.99) [121.4]	−0.02 (−0.48, 0.44) [−5682.9]	0.436

IL-6, interleukin-6; TNF-α, tumor necrosis factor-α; hs-CRP, high-sensitivity C-reactive protein; CI, confidence interval; CV, coefficient of variation.

Hand grip strength: Maximum hand grip strength of the right and left hands; Absolute knee extensor strength: a maximum external torque of the quadriceps muscles; Absolute knee flexor strength: a maximum flexor strength of the quadriceps muscles; Usual gait speed: a test to measure the speed that a person is able to walk over a specific distance on a level surface; Timed up and go test: a test to quantifies functional mobility, lower-leg muscle strength, and gait performance; Short physical performance battery: a test to measure functional strength and physical function in the lower extremities.

1 Significant difference between baseline and 12 weeks data by paired t test at * *p* < 0.05, ** *p* < 0.01, *** *p* < 0.001.

2 Significant difference in changes between baseline and 12 weeks by student t-test.

3 Significant difference in changes between baseline and 12 weeks by ANCOVA adjusted for physical activity level at * *p* < 0.05, ** *p* < 0.01, *** *p* < 0.001.

## 4 Discussion

The placebo-controlled study observed the effects of the protein-enriched ONS on muscle function in females aged 50–80 years for 12 weeks. Throughout the study, participants maintained their usual physical activity levels, and despite ONS supplementation, total energy intake remained unchanged from baseline. When consumed alongside meals, the protein-enriched ONS, which contained BCAAs and trace nutrients, contributed to a reduction in body fat and an increase in lean body mass.

The fundamental requirement that influences protein synthesis and skeletal muscle mass is dietary protein [16,29,30]. Protein supplementation or a high-protein diet suppresses the reduction of muscle mass and muscle strength and increases muscle fiber [[31], [32], [33], [34], [35]]. Especially, BCAAs are known to help prevent muscle loss and promote muscle mass gain [36]. Loenneke et al. found that consuming meals containing 30–45 g of protein per meal was associated with increases in leg lean body mass and strength [37]. Many studies reporting positive effects of protein supplementation on sarcopenia, similar to this study, administered 1.3–1.5 g/kg of protein over 12 weeks [[38], [39], [40], [41]]. Ten Haaf et al. reported an increase in lean body mass and a decrease in body fat when supplementing with milk protein at approximately 1.3 g/kg/day over 12 weeks [38]. Similarly, Park, et al. [39], Griffen, et al. [40], and Nakayama, et al. [41], also observed improvements in lean body mass or skeletal muscle mass when supplementing with milk protein at around 1.5 g/kg/day. It can be seen that protein supplementation contributed to the improvement of the quantity and quality of the subjects' muscles. In our study, both groups demonstrated adequate protein intake at baseline, exceeding the recommended intake level for Korean older adults (0.91 g/kg/d, based on the 2020 Korean Dietary Reference Intakes) [42]. After 12 weeks, while protein intake decreased in the placebo group, it remained within the recommended range, whereas the test group’s protein intake increased to 1.5 g/kg/d. These findings suggest that the observed effects may have been influenced not only by the slight reduction in protein intake in the placebo group, which remained within the recommended range, but also by the increase in protein intake in the test group, reaching approximately 1.5 g/kg/d.

On the contrary, there have been claims suggesting that excessive protein intake may increase the risk of sarcopenia. According to a cross-sectional study using a twin cohort in the United Kingdom, high protein intake of 1.3 g/kg per day was reported to be associated with sarcopenia [43]. However, this result represents a correlation that cannot explain causality, so it does not exclude the possibility that individuals with sarcopenia may already be consuming high-protein diets for therapeutic purposes. Furthermore, given that the primary protein sources for participants in this United Kingdom study were mostly animal-based foods such as meat, there is a possibility of developing metabolic acidosis, which could impair kidney function and have detrimental effects on muscles. However, further research is needed to investigate this possibility [43,44].According to the study by Griffen et al., protein intake up to 1.5 g/kg/day did not have a negative impact on renal function [40]. Similar to our study, it appears that a daily protein intake level of around 1.5 g/kg does not have a significant impact on muscle loss and other side effects.

ONS can be an effective strategy for supplementing deficient or missed nutrients to prevent sarcopenia. As a result of the food record analysis in this study, after ONS intervention, the intake of protein, vitamin A, vitamin D, vitamin E, calcium, phosphorus, and zinc significantly increased in the test group. These results are consistent with previous studies showing that ONS consumption increases the intake of protein, vitamins, and minerals in older adults [45]. However, in this study, although participants were instructed to maintain their usual diet and consume ONS as a supplement, the consumption of ONS naturally led to a decrease in food intake. Nevertheless, it allowed participants to meet the required intake of nutrients such as protein, vitamins, and minerals without an excessive increase in energy intake. This finding was also observed in the study by Abizanda et al. [46], which involved a 12-week intervention including both ONS supplementation and exercise in older adults. Although participants were instructed to maintain their usual diet while consuming ONS as a supplement, there was no significant difference in total energy intake before and after the intervention, similar to our study. Despite the reduction in habitual food intake, the intake of protein, vitamin A, vitamin D, vitamin E, calcium, phosphorus, and zinc significantly increased, contributing to improvements in physical function, nutritional status, and quality of life in older adults. We highlight, however, that ONS should not be regarded as a strict replacement for meals. Rather, it should be recognized as a valuable option for individuals with inadequate dietary intake who require supplementation to meet their nutritional needs.

Muscle strength, physical performance, and inflammatory markers did not show significant differences between the two groups. Since muscle strength and physical performance are directly linked to physical activity, the relatively modest results could be attributed to the lack of intervention targeting physical activity. In the study by Lochlainn et al., after adjusting for confounding factors such as age and sex, it was found that there was no significant association between protein intake and muscle strength [43]. For physical activity levels, although the test group had higher levels than the placebo group at baseline, there was no difference in physical activity levels between the two groups after 12 weeks. The age distribution of participants was also similar, with the majority in their 60 s, indicating that the baseline difference in physical activity levels was not attributable to differences in age distribution. Furthermore, according to the IPAQ classification, both groups fell within the moderate physical activity level range (600–3000 MET-min/week) at baseline and after 12 weeks, suggesting that the observed differences were not clinically meaningful [47,48]. Therefore, it can be concluded that the increase in lean body mass and decrease in body fat over the 12-week period were outcomes of ONS supplementation that were not significantly influenced by the level of physical activity.

The present study has several strengths. Participants demonstrated a high compliance rate of over 95%, indicating excellent adherence, despite the requirement for participants to consume ONS twice a day throughout the study period. Additionally, it is noteworthy that middle-aged and elderly women experienced improvements in lean body mass and reduction in body fat mass without any reported side effects or the aid of medication.

Despite these strengths, this study has several limitations. First, it is difficult to generalize the results of the study to represent all females aged 50–80 years as the study subjects were recruited from only volunteers from a specific region. Second, this study involved postmenopausal females with no impairment in activities of daily living and a low risk of disease, whose basic nutritional status and muscle mass and function may not have been sufficiently compromised to benefit from an ONS intake. Further interventional studies using vulnerable subjects such as low handgrip strength or malnutrition or involving exercise interventions are needed.

In conclusion, protein-enriched ONS containing BCAAs had a positive effect on improving nutritional status. Furthermore, ONS demonstrated an improvement in body composition by increasing lean body mass and reducing fat mass. These changes in body composition are expected to have a long-term effect on preventing sarcopenia and improving muscle health according to the natural decrease in muscle mass due to aging.

## CRediT authorship contribution statement

Concept and design: Y Lim, J Chon, H Lim.

Acquisition, analysis, or interpretation of data: H Rho, M Kim, M Lee.

Statistical analysis: H Rho, M Kim, M Lee.

Drafting of the manuscript: M Kang, H Rho.

Review and editing of the manuscript: M Kang, H Lim.

Methodology: Y Lim, J Chon, H Lim.

Supervision: Y Lim, J Chon, H Lim.

## Funding

This work was supported by the Daesang Wellife Corp. (Seoul, Republic of Korea) [CRIS No. KCT0007708].

## Declaration of competing interest

The authors declare that they have no conflicts of interest in this work.

## References

[1] Morley J.E., Baumgartner R.N., Roubenoff R., Mayer J., Nair K.S. (2001). Sarcopenia. J Lab Clin Med..

[2] Cruz-Jentoft A.J., Baeyens J.P., Bauer J.M., Boirie Y., Cederholm T., Landi F. (2010). Sarcopenia: European consensus on definition and diagnosis: report of the European Working Group on Sarcopenia in Older People. Age Ageing..

[3] Talbot J., Maves L. (2016). Skeletal muscle fiber type: using insights from muscle developmental biology to dissect targets for susceptibility and resistance to muscle disease. Wires Dev Biol..

[4] Koster A., Ding J., Stenholm S., Caserotti P., Houston D.K., Nicklas B.J. (2011). Does the amount of fat mass predict age-related loss of lean mass, muscle strength, and muscle quality in older adults?. J Gerontol A Biol Sci Med Sci..

[5] Larsson L., Degens H., Li M., Salviati L., Lee Y.I., Thompson W. (2019). Sarcopenia: aging-related loss of muscle mass and function. Physiol Rev..

[6] Ooi H., Welch C. (2024). Obstacles to the early diagnosis and management of sarcopenia: current perspectives. Clin Interv Aging..

[7] Hwang J., Park S. (2024). Korean nationwide exploration of sarcopenia prevalence and risk factors in late middle-aged women. Healthcare (Basel)..

[8] Pellegrino A., Tiidus P.M., Vandenboom R. (2022). Mechanisms of estrogen influence on skeletal muscle: mass, regeneration, and mitochondrial function. Sports Med..

[9] Maltais M.L., Desroches J., Dionne I.J. (2009). Changes in muscle mass and strength after menopause. J Musculoskelet Neuronal Interact..

[10] Scott D., Jones G. (2014). Impact of nutrition on muscle mass, strength, and performance in older adults. Osteoporosis Int..

[11] Vermeulen A., Goemaere S., Kaufman J.M. (1999). Testosterone, body composition and aging. J Endocrinol Invest..

[12] Thorner M.O., Nass R. (2007). Human studies of growth hormone and aging. Pediatr Endocrinol Rev..

[13] Baumgartner R.N., Waters D.L., Gallagher D., Morley J.E., Garry P.J. (1999). Predictors of skeletal muscle mass in elderly men and women. Mech Ageing Dev..

[14] Kim T.N., Choi K.M. (2013). Sarcopenia: definition, epidemiology, and pathophysiology. J Bone Metab..

[15] Liguori I., Russo G., Aran L., Bulli G., Curcio F., Della-Morte D. (2018). Sarcopenia: assessment of disease burden and strategies to improve outcomes. Clin Interv Aging..

[16] Paddon-Jones D., Rasmussen B.B. (2009). Dietary protein recommendations and the prevention of sarcopenia. Curr Opin Clin Nutr Metab Care..

[17] Houston D.K., Nicklas B.J., Ding J., Harris T.B., Tylavsky F.A., Newman A.B. (2008). Dietary protein intake is associated with lean mass change in older, community-dwelling adults: the Health, Aging, and Body Composition (Health ABC) study. Am J Clin Nutr..

[18] Saito K., Yokoyama T., Yoshida H., Kim H., Shimada H., Yoshida Y. (2012). A significant relationship between plasma vitamin C concentration and physical performance among Japanese elderly women. J Gerontol A Biol Sci Med Sci..

[19] Annweiler C., Schott A.M., Berrut G., Fantino B., Beauchet O. (2009). Vitamin D-related changes in physical performance: a systematic review. J Nutr Health Aging..

[20] Chung E., Mo H., Wang S., Zu Y., Elfakhani M., Rios S.R. (2018). Potential roles of vitamin E in age-related changes in skeletal muscle health. Nutr Res..

[21] Hagstromer M., Oja P., Sjostrom M. (2006). The International Physical Activity Questionnaire (IPAQ): a study of concurrent and construct validity. Public Health Nutr..

[22] Trampisch U.S., Franke J., Jedamzik N., Hinrichs T., Platen P. (2012). Optimal jamar dynamometer handle position to assess maximal isometric hand grip strength in epidemiological studies. J Hand Surg-Am..

[23] Kim M., Won C.W. (2019). Combinations of gait speed testing protocols (automatic vs manual timer, dynamic vs static start) can significantly influence the prevalence of slowness: results from the Korean Frailty and Aging Cohort Study. Arch Gerontol Geriat..

[24] Guralnik J.M., Simonsick E.M., Ferrucci L., Glynn R.J., Berkman L.F., Blazer D.G. (1994). A short physical performance battery assessing lower-extremity function - association with self-reported disability and prediction of mortality and nursing-home admission. J Gerontol..

[25] Greene B.R., O’Donovan A., Romero-Ortuno R., Cogan L., Scanaill C.N., Kenny R.A. (2010). Quantitative falls risk assessment using the timed up and go test. IEEE T Bio-Med Eng..

[26] Kim H.K., Suzuki T., Saito K., Yoshida H., Kobayashi H., Kato H. (2012). Effects of exercise and amino acid supplementation on body composition and physical function in community-dwelling elderly Japanese sarcopenic women: a randomized controlled trial. J Am Geriatr Soc..

[27] Studenski S.A., Peters K.W., Alley D.E., Cawthon P.M., McLean R.R., Harris T.B. (2014). The FNIH sarcopenia project: rationale, study description, conference recommendations, and final estimates. J Gerontol a-Biol.

[28] Baek J.Y., Jung H.W., Kim K.M., Kim M., Park C.Y., Lee K.P. (2023). Korean Working Group on Sarcopenia Guideline: expert consensus on sarcopenia screening and diagnosis by the Korean society of sarcopenia, the Korean society for bone and mineral research, and the Korean geriatrics society. Ann Geriatr Med Res..

[29] Paddon-Jones D., Sheffield-Moore M., Katsanos C.S., Zhang X.J., Wolfe R.R. (2006). Differential stimulation of muscle protein synthesis in elderly humans following isocaloric ingestion of amino acids or whey protein. Exp Gerontol..

[30] Paddon-Jones D., Sheffield-Moore M., Zhang X.J., Volpi E., Wolf S.E., Aarsland A. (2004). Amino acid ingestion improves muscle protein synthesis in the young and elderly. Am J Physiol Endocrinol Metab..

[31] Nilsson A., Montiel Rojas D., Kadi F. (2018). Impact of meeting different guidelines for protein intake on muscle mass and physical function in physically active older women. Nutrients..

[32] Kim I.Y., Schutzler S., Schrader A., Spencer H., Kortebein P., Deutz N.E. (2015). Quantity of dietary protein intake, but not pattern of intake, affects net protein balance primarily through differences in protein synthesis in older adults. Am J Physiol Endocrinol Metab..

[33] Granic A., Mendonca N., Sayer A.A., Hill T.R., Davies K., Adamson A. (2018). Low protein intake, muscle strength and physical performance in the very old: the Newcastle 85+ Study. Clin Nutr..

[34] McLean R.R., Mangano K.M., Hannan M.T., Kiel D.P., Sahni S. (2016). Dietary protein intake is protective against loss of grip strength among older adults in the framingham offspring cohort. J Gerontol A Biol Sci Med Sci..

[35] Kobayashi S., Asakura K., Suga H., Sasaki S., Three-generation Study of Women on D, Health Study G (2013). High protein intake is associated with low prevalence of frailty among old Japanese women: a multicenter cross-sectional study. Nutr J..

[36] Ooi D.S.Q., Ling J.Q.R., Ong F.Y., Tai E.S., Henry C.J., Leow M.K.S. (2021). Branched chain amino acid supplementation to a hypocaloric diet does not affect resting metabolic rate but increases postprandial fat oxidation response in overweight and obese adults after weight loss intervention. Nutrients..

[37] Loenneke J.P., Loprinzi P.D., Murphy C.H., Phillips S.M. (2016). Per meal dose and frequency of protein consumption is associated with lean mass and muscle performance. Clin Nutr..

[38] Ten Haaf D.S.M., Eijsvogels T.M.H., Bongers C., Horstman A.M.H., Timmers S., de Groot L. (2019). Protein supplementation improves lean body mass in physically active older adults: a randomized placebo-controlled trial. J Cachexia Sarcopenia Muscle..

[39] Park Y., Choi J.E., Hwang H.S. (2018). Protein supplementation improves muscle mass and physical performance in undernourished prefrail and frail elderly subjects: a randomized, double-blind, placebo-controlled trial. Am J Clin Nutr..

[40] Griffen C., Duncan M., Hattersley J., Weickert M.O., Dallaway A., Renshaw D. (2022). Effects of resistance exercise and whey protein supplementation on skeletal muscle strength, mass, physical function, and hormonal and inflammatory biomarkers in healthy active older men: a randomised, double-blind, placebo-controlled trial. Exp Gerontol..

[41] Nakayama K., Saito Y., Sanbongi C., Murata K., Urashima T. (2021). Effects of low-dose milk protein supplementation following low-to-moderate intensity exercise training on muscle mass in healthy older adults: a randomized placebo-controlled trial. Eur J Nutr..

[42] Ministry of Health and Welfare, The Korean Nutrition Society (2020).

[43] Ni Lochlainn M., Bowyer R.C.E., Welch A.A., Whelan K., Steves C.J. (2023). Higher dietary protein intake is associated with sarcopenia in older British twins. Age Ageing..

[44] Welch A.A., MacGregor A.J., Skinner J., Spector T.D., Moayyeri A., Cassidy A. (2013). A higher alkaline dietary load is associated with greater indexes of skeletal muscle mass in women. Osteoporos Int..

[45] Milne A.C., Potter J., Vivanti A., Avenell A. (2009). Protein and energy supplementation in elderly people at risk from malnutrition. Cochrane Database Syst Rev..

[46] Abizanda P., Lopez M.D., Garcia V.P., Estrella Jde D., da Silva Gonzalez A., Vilardell N.B. (2015). Effects of an Oral nutritional supplementation plus physical exercise intervention on the physical function, nutritional status, and quality of life in frail institutionalized older adults: the ACTIVNES study. J Am Med Dir Assoc..

[47] Zhou Z., Tian X. (2024). Prevalence and association of sleep duration and different volumes of physical activity with type 2 diabetes: the first evidence from CHARLS. BMC Public Health..

[48] Craig C.L., Marshall A.L., Sjostrom M., Bauman A.E., Booth M.L., Ainsworth B.E. (2003). International physical activity questionnaire: 12-country reliability and validity. Med Sci Sports Exerc..

